# On the Remedies Used to Promote Absorption of Inflammatory and Other Morbid Products

**Published:** 1882-01

**Authors:** 


### ON THE REMEDIES USED TO PROMOTE ABSORPTION OF INFLAMMATORY AND OTHER MORBID PRODUCTS.

*International Medical Congress.*

Dr. Dujardin Beaumetz read a paper on this subject. He said that, rightly to estimate the value of therapeutic interference in the circumstances indicated, the changes that normally occurred without it must first be considered. The living cell was the theatre of a continual integration and disintegration, the materials for which were supplied by the blood and lymph. Under irritation it only underwent visible alterations, often in the direction of inordinate development. After a time, however, this pathological activity usually came to a stop, and the abnormal elements produced might gradually disappear; first, by the removal of corpuscular elements, still in an early stage of organization, by the lymphatic circulation; secondly, by fatty or granular degeneration of the newly formed elements, and the subsequent absorption of the detritus thus produced; lastly, by fibroid elements taking the place of the embryonic tissue, and checking its further development. Sometimes, in the case of serous and mucous membranes, sero-fibrinous effusions were poured out, and were reabsorbed by the capillaries, either unchanged or after undergoing fatty-granular metamorphosis. Lastly, the influence of the nervous system in controlling tissue metabolism must not be left out of account. Therapeutic interference might, to a certain extent, promote the absorption of these morbid and inflammatory products. Medicines designed to favor it had been termed "resolvent." The old pharmacopœia divided them into "solvent," "deobstruent" and "absorbent" remedies; the first intended to soften the morbid products; the second to facilitate the circulation through the capillary and lymphatic vessels; the third to promote the absorption of the products after they had undergone more or less profound changes. This old classification furnished a very real explanation of the phenomena. To hasten absorption, it was necessary to assist the processes which normally occurred in the diseased tissues; to hasten their return to their embryonic phase, or their fatty-granular degeneration, or the development of the fibroid cicatricial tissue which tended to strangle them. The remedial measures for the promotion of these objects were manifold. Some acted mechanically (compression, massage, etc.); others, of a more active kind, by evulsion (blisters, "solvent" ointments and plasters). Other remedial agents acted still more directly on nutrition, through the nervous or the vaso-motor system (the continuous galvanic current, applied to the surface, or by electro puncture). A destructive action on the morbid products, designed to promote their degeneration and absorption, had been aimed at in other ways (injections of Corsica *papaya* or *papayin* into malignant tumors; toxic injections to destroy the fœtus in extra-uterine pregnancy, etc.). Again, the liquid products of inflammation might sometimes be removed by surgical means (pus, sero-fibrinous effusions, etc.). There remained to be considered those medicaments, in the strict sense of the term, which had a solvent action on inflammatory and other morbid products. First, however, the influence of the general nutrition of the body on the nutrition of such product deserved notice. Both augmentation and diminution of nutritive interchange exerted such an influence. The former might cause morbid products to be absorbed by stimulating the functions of the economy (*e. g.*, removal of strumous and lymphatic deposits by appropriate hygienic measures---country air, nourishing food, regulated exercises; these measures increasing the activity of the capillary and lymphatic circulation). Abstinence and diseases productive of mal-nutrition might achieve the same end in another fashion (autophagy of muscular and adipose tissue; similar action on tumors chiefly chiefly made up of fat). A like method had been proposed with a view to the removal of inflammatory exudations into serous cavities (peritoneum, pleura). Apart from medicines which acted on certain inflammatory exudations (*e. g.*, diuretics and purgatives in pleuritic and peritoneal effusions), and from such as operated by affecting nutrition (*e. g.*, arsenic), there were only two drugs which exerted a real and selective action on the nutrition of such new formations; iodine (including iodides) and mercury. Clinical experience afforded daily proof of their value; experimental research had not yet explained it. These remedies had a selective power over certain products, and not over others which might appear to be structurally identical to them. Thus, syphilitic gummata, strumous deposits, and tubercles were histologically similar; but mercury only affected the first, iodine the second, while the progress of the third was in no degree influenced by either of the two medicaments. Hemlock and its preparations were formerly in great repute as solvents; notwithstanding the discovery of conia, however, there was still no explanation of this special action of hemlock. It might possibly be due to its influence upon the nervous system. Its true value in this respect deserved further clinical investigation.

